# Ultrasound-Assisted Extraction of Polyphenols from Maritime Pine Residues with Deep Eutectic Solvents

**DOI:** 10.3390/foods11233754

**Published:** 2022-11-22

**Authors:** Hugo Duarte, Valentim Gomes, María José Aliaño-González, Leonor Faleiro, Anabela Romano, Bruno Medronho

**Affiliations:** 1 MED—Mediterranean Institute for Agriculture, Environment and Development, Faculdade de Ciências e Tecnologia, Campus de Gambelas, Universidade do Algarve, Ed. 8, 8005-139 Faro, Portugal; 2 Departamento de Química Analítica, Facultad de Ciencias, Universidad de Cádiz, 11510 Cadiz, Spain; 3 Champalimaud Research Program, Champalimaud Centre for the Unknown, 1400-038 Lisbon, Portugal; 4 Faculdade de Ciências e Tecnologia, Campus de Gambelas, Universidade do Algarve, Ed. 8, 8005-139 Faro, Portugal; 5 Algarve Biomedical Center, Research Institute, 8005-139 Faro, Portugal; 6 FSCN—Fibre Science and Communication Network Research Center, Surface and Colloid Engineering, Mid Sweden University, SE-851 70 Sundsvall, Sweden

**Keywords:** Box–Behnken experimental design, *Pinus pinaster*, biomass, deep eutectic solvents, green solvents, antimicrobial activity, antioxidant activity

## Abstract

Deep eutectic solvents represent an important alternative in the field of green solvents due to their low volatility, non-toxicity, and low synthesis cost. In the present investigation, we propose the production of enriched polyphenolic extracts from maritime pine forest residues via an ultrasound-assisted approach. A Box–Behnken experimental design with a response surface methodology was used with six variables to be optimized: solid-to-solvent ratio, water percentage, temperature and time of extraction, amplitude, and catalyst concentration. The mixture of levulinic and formic acids achieved the highest extraction yield of polyphenols from pine needle and bark biomass. In addition, the solid-to-solvent ratio was found to be the only influential variable in the extraction (*p*-value: 0.0000). The optimal conditions were established as: 0.1 g of sample in 10 mL of LA:FA (70:30%, *v*/*v*) with 0% water and 0 M H_2_SO_4_ heated to 30 °C and extracted during 40 min with an ultrasound amplitude of 80% at 37 kHz. The bioactive properties of polyphenol-enriched extracts have been proven with significant antioxidant (45.90 ± 2.10 and 66.96 ± 2.75 mg Trolox equivalents/g dw) and antimicrobial activities. The possibility to recycle and reuse the solvent was also demonstrated; levulinic acid was successfully recovered from the extracts and reused in novel extractions on pine residues. This research shows an important alternative to obtaining polyphenol-enriched extracts from forest residues that are commonly discarded without any clear application, thus opening an important window toward the valorization of such residues.

## 1. Introduction

Maritime pine (*Pinus pinaster* Aiton) forests account for approximately 23% of Portuguese forests, providing a significant amount of harvested wood as raw material for the wood-based industries [1]. Consequently, a huge amount of pine-based residues are annually produced. These have been explored as potential renewable energy sources, with several recent public policies launched to encourage the use of residual forest biomass in Portugal [2]. However, maritime pine biomass holds many different chemicals, with added-value potential suitable for several applications [3]. Being a rich source of polyphenols with antioxidant and antimicrobial properties, the maritime pine extracts offer great potential for therapeutical practices, food enrichment, and biomaterial applications [4,5]. However, in order to produce extracts suitable for such applications, suitable solvents and extraction methodologies must be employed, according to the matrix source and desired type of target molecules [6].

Different methods have been developed ranging from the conventional maceration, Soxhlet, and solid–liquid extractions, to the more advanced supercritical fluid, pressurized water, microwave, or ultrasound-based approaches, which have been extensively performed in many different types of biomass [3,7,8]. Associated with these, harsh organic solvents have always been used for the extraction of bioactive compounds [9,10]. However, most modern techniques, such as ultrasound-assisted extraction (USE), are more efficient than conventional ones, mainly by reducing solvent volume, extraction time, and associated costs.

USE is based on the propagation of ultrasound pressure waves, resulting in cavitation forces that induce the explosive collapse of bubbles. This, generates localized pressure regions causing plant tissue rupture and improving the release of intra-cellular compounds to the solvent [11,12]. Nonetheless, advanced techniques such as USE also require optimization, mainly regarding solvent selection.

Nowadays, with the increasing environmental concern, some newly developed solvents have successfully replaced the commonly used organic solvents, such as methanol or acetone, in the extraction of bioactive components used in the pharmaceutical, food, and cosmetic industries. This is the case of deep eutectic solvents (DES), systems that are formed by mixing an appropriate molar ratio of a hydrogen-bond acceptor (HBA) with a hydrogen-bond donor (HBD) [13,14,15]. Once formed, these homogeneous mixtures present a significantly lower melting point than the melting points of each component [16,17]. These new solvent systems display similar properties to ionic liquids (ILs). In addition to the low volatility, DES are also non-toxic and simple to prepare at a lower cost than the traditional ILs. Contrary to ILs, DES synthesis and regeneration does not imply any chemical reaction as it should mainly involve the formation and rupturing of the hydrogen bonding network [16,18]. In certain cases, DESs can also hydrate up to 50% (*v*/*v*) without compromising their dissolution performance [19]. In fact, water addition has proved to influence the DES properties, such as density, viscosity, conductivity, and polarity [20]. The addition of water is desirable since most DESs have high viscosity. Therefore, the efficiency of extraction can be enhanced by improving the mass transfer rate by lowering the viscosity [21].

DESs have been used in the biomass fractionation of different plant species, such as acacia, *Thymus*, palm, *Lavandula*, or rosemary; fruit waste from olive, orange, pear, and grape skin; forest biowastes as pine needles and bark, wood from different trees or even the industrially processed kraft pulp; adding to this animal-based wastes such as cod skin and wool [12,14,17,22,23,24,25]. These are only a few examples of biomass versatility where DES-assisted extractions have been successfully employed. The ability to obtain valuable phenolic compounds from the valorization of bioresources and their application, for instance, in medicine, food, and cosmetics, is a key factor for a bio-based and sustainable economy [26]. Following this motto, in this research, the ultrasonic-assisted extraction of polyphenols present in pine needle and bark residues was assessed using several solvent systems. Choline chloride-based DES, acid and alkali solvents, and also a biomass-derived binary solvent system composed of levulinic acid:formic acid (LA:FA), previously used for lignin extraction from pine sawdust [27], were compared.

Since there are many variables related to the USE or the DESs preparation that can affect the extraction of polyphenols, a reliable design of experiment is required to evaluate the influence of each variable, their possible interactions, and consequently, their optimization to achieve the highest yield of extracted polyphenols. In this regard, a demanding Box–Behnken design with response surface methodology was employed to evaluate the influence of the different variables related to the extraction methodology on the final polyphenols’ recovery and bioactive properties. To the best of our knowledge, this is the first time that DESs combined with the USE technique have been employed for the extraction of polyphenols from maritime pine residues.

## 2. Materials and Methods

### 2.1. Chemicals

The solvents levulinic acid (LA), lactic acid (Lact), formic acid (FA), and acetic acid (Act) were acquired from Merck (Darmstadt, Germany), whereas choline chloride (ChCl) and methanol (MeOH) were purchased from Thermo Fisher Scientific (Waltham, MA, USA). Sodium hydroxide (NaOH), potassium hydroxide (KOH), and sulfuric acid (H_2_SO_4_) were obtained from Panreac (Barcelona, Spain). All solvents and reagents were purchased in analytical grade. When required, distilled-grade water was used.

Different solutions were prepared to evaluate the efficiency of polyphenols extraction from *P. pinaster* residues: (i) LA:H_2_O (30:60, *v*/*v*); (ii) LA:FA (70:30, *v*/*v*); (iii) LA:ChCl (70:30, *v*/*v*); (iv) Lact:ChCl (70:30, *v*/*v*); and (v) a solution of MeOH:H_2_O (80:20, *v*/*v*), solutions of NaOH:H_2_O (1:99, *v*/*v*) and KOH:H_2_O (1:99, *v*/*v*), and solutions of Act:H_2_O (24:76, *v*/*v*) and Lact:H_2_O (36:64, *v*/*v*) were used as standard chemical solvents. Solutions (ii), (iii), and (iv) were subsequently diluted in 60% of water: LA/FA:H_2_O (30:60, *v*/*v*), LA/ChCl:H_2_O (30:60, *v*/*v*), Lact/ChCl:H_2_O (30:60, *v*/*v*). These were prepared by manual mixing, followed by stirring to ensure the complete homogenization of the final solution.

Gallic acid (95% purity, Sigma-Aldrich Chemical Co., St. Louis, MO, USA) was used as standard to quantify the total polyphenol content. For the antioxidant activity measurements, 2,2-diphenyl-1-picrylhydrazyl (DPPH) from Sigma-Aldrich (St. Louis, MO, USA), and 6-hydroxy-2,5,7,8-tetramethylchroman-2-carboxylic acid (Trolox) from Thermo Fisher Scientific (Waltham, MA, USA) were used as standards. Cultures of *S. aureus* (ATCC 6538) were grown on Tryptic soy broth from Biokar Diagnostics (Paris, France).

### 2.2. Samples

Pine residues (needles and bark) were collected from *P. pinaster* trees in the vicinity of the Universidade do Algarve in the Campus of Gambelas (Gambelas, Faro, Portugal) in October of 2021. Dirt, insects, and possible chemical residues were removed by washing with deionized water. After that, residues were manually cut into small pieces and dried in an oven (Model ED23, Binder GmbH, Tuttlingen, Germany) at 40 °C until constant weight, achieving 5% of moisture. Finally, the biomass was pulverized in a grinder until obtaining a fine and uniform powder that was kept dry at room temperature until further analysis.

### 2.3. Polyphenols Extraction

#### 2.3.1. Ultrasound-Assisted Extraction System

Ultrasound-assisted extractions were performed using a 5 mm two-cylinder ultrasound probe coupled to a Vibracell 75186 ultrasound (Sonics, Newton, UK) (20 kHz, 130 W). For each extraction assay, 1.5 g of the sample was weighed in an Erlenmeyer flask with a capacity of 100 mL, and 10 mL of the corresponding solvent was added. The sample was placed in a water bath at the desired controlled temperature with a thermometer control unit. The ultrasound probe was carefully introduced without touching the bottom of the Erlenmeyer, and the frequency was fixed at 37 kHz. The corresponding amplitude and time were established before performing the extractions. After that, extracts were filtered with a Buchner funnel and a vacuum pump, collected in 10 mL volumetric flasks and kept at 5 °C until further analysis.

#### 2.3.2. Box–Behnken Design of Experiments

The design of experiments is a methodology that relies on a mathematical description of the system behavior (in this case, polyphenols extraction employing USE) using the minimal number of experiments [28]. There are many designs of experiments, such as full factorial design, central composite design, mixture design, etc. In this case, a Box–Behnken design with response surface methodology (BBD-RSM) was used for the optimization of polyphenols extraction from pine residues. In this kind of design, three levels are assigned to each factor: (−1) as the lowest level, (0) as the intermediate level, and (1) as the highest level, producing a cube that consists of a central point and middle points at the edges, avoiding axial points [29]. It should be highlighted that in this approach, the number of experiments required to achieve the same information is lower, reason why it is considered more efficient than many of the alternative three-level full factorial designs [30]. In addition, the experiments performed avoid extreme conditions, so experimental methods that may pose an excessive economic or power consumption demand or that might lead to the degradation of the polyphenols are discarded [31].

The BBD-RSM can be defined by the following mathematical relation:

(1) $$Y=\beta_{0}+\sum_{i=1}^{3}\beta_{i}X_{i}+\sum_{i=1}^{3}\beta_{ii}X_{ii}^{2}+\sum_{i=1}^{2}\sum_{j=i+1}^{3}\beta_{ij}X_{i}X_{j}$$

where *Y* is the response variable (i.e., total polyphenols); and *β*_0_, *β_i_*, *β_ii_*, *β_ij_* are the regression coefficients of the intercept, linear, quadratic and interaction terms, respectively. *X_i_* and *X_j_* are the independent variables.

Six variables were selected to be optimized: solid-to-solvent ratio (0.50–2.50 g/10 mL of solvent), water percentage (0–60%), temperature of extraction (30–60 °C), time of extraction (1–2 h), amplitude (20–80%), and catalyst concentration (0–2 M H_2_SO_4_). These variables and the studied range were based on the previous experience of the research group [17,27,32,33]. It is important to remark that although DESs mixtures usually present high boiling points due to their inherent physicochemical characteristics, many authors have proved the degradation of polyphenols at temperatures higher than 60 °C [34]. Thus, in this work, the maximum temperature was limited to 60 °C.

#### 2.3.3. Total Polyphenols Content

The optimization of the USE method was based on extracting the maximum total polyphenols from pine residues. The extracts were analyzed at 280 nm, using a Genesys 10uv spectrophotometer (Thermo Scientific, Waltham, MA, USA), a wavelength where maximum absorbance is observed in polyphenols [35]. A gallic acid calibration curve was prepared between 0 and 800 mg/L and used as standard to estimate the total concentration of polyphenols. The calibration curve obtained was y = 6.08x − 0.01, where y is the absorbance, and x is the gallic acid concentration in mg/L. The coefficient of regression was R^2^ = 0.9994. The total polyphenol concentration was expressed as mg/g dry weight (dw).

### 2.4. Antioxidant Activity

The DPPH method was selected to evaluate the antioxidant activity of the extracts [36]. The procedure consisted of mixing 300 µL of DPPH solution [90 µM DPPH in methanol:H_2_O (80:20% *v*/*v*)] with 30 µL of the extract and 570 µL of the MeOH:H_2_O solution (80:20% *v*/*v*). This mixture was kept at room temperature and in the dark for 30 min, and the absorbance was measured at 515 nm. Trolox was the selected standard, and a six-point regression curve was prepared with concentrations from 0 mM to 0.30 mM. The obtained equation was y = 221.57x + 2.51, with a coefficient of R^2^ = 0.9915. The antioxidant activity was expressed as mg of Trolox equivalents (TE) per gram of dry weight (mg TE/g dw).

### 2.5. Antimicrobial Activity

The antimicrobial activity of the extracts was evaluated following the *S. aureus* (ATCC 6538) growth in the presence of the extracted polyphenols. The initial inoculum had a concentration of 10^4^–10^5^ colony-forming units (CFU/mL: colony-forming units per milliliter), grown in TSB (Tryptic soy broth) medium. The following procedure considers 200 µL of the inoculum, which was loaded in microplate wells and supplemented with 10 µL of the polyphenol extract. All tests were performed in triplicate, and a non-supplemented bacterial culture was used as a control. After 24 h at 37 °C, the optical density of the culture was measured at 600 nm, and the bacterial growth was evaluated.

### 2.6. Solvent Reuse

As previously mentioned, DESs mixtures can be considered “green solvents” due to their inherent physicochemical characteristics, origin, and synthesis procedure. For this reason, the recycling and reuse of the DES mixture employed for the polyphenols’ extraction were evaluated. The extracts obtained from pine residues under optimal conditions were diluted 1:100 with deionized water and filtered using a Strata-X cartridge (Phenomenex, Madrid, Spain). The polyphenols were retained in the cartridge, and the diluted solvent was collected. Those extracts were analyzed by LC-MS to ensure that no LA and FA were present. The water and FA were evaporated using a rotary evaporator fixed at 100 °C. After 15 min, the recovered LA was collected, reused for the preparation of novel LA:FA (70%, 30% *v*/*v*) mixtures, and applied in subsequent pine needle and bark extractions under optimal conditions.

### 2.7. Data Analysis

The BBD-RSM design was performed using Statgraphic Centurion software (version XVII) (Statgraphics Technologies, Inc., The Plains, VA, USA). During the analysis of the results, data were compared and grouped according to the LSD (least significant difference), response surface regression techniques, and the Fisher test considering a significance of 95% (*p*-value ≤ 0.05).

## 3. Results and Discussion

### 3.1. Suitability of DESs Solvents

The first task of this research focused on the feasibility of using the DESs mixtures for the extraction of polyphenols from pine residues. To do so, different extractions were carried out using the solvent mixtures and experimental conditions introduced in the experimental section.

**Figure 1:**
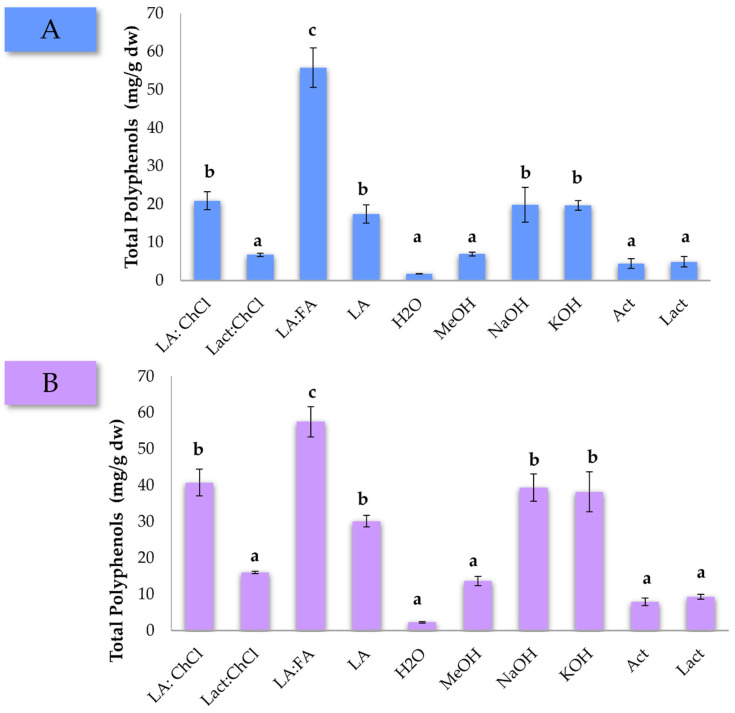
Total polyphenols concentration (mg/g dw) for *P. pinaster* (**A**) needles and (**B**) bark according to the different DES mixtures and chemical solvents used for the extraction. Solvent acronyms have been defined in the experimental section. Different letters indicate significant differences in the ANOVA Duncan post hoc test with 95% of confidence.

The extractions were carried out for 1 h in triplicate, and the followed procedure to obtain the final extracts can be found in the Section 2. The average of the total polyphenol concentration is represented in Figure 1. The first striking observation is that the LA:FA mixture allowed extracting the maximum concentration of polyphenols in both pine matrices. Moreover, the LA:ChCl mixture is regarded as an important alternative for the extraction of these compounds, reaching higher concentrations than those extracted with NaOH and KOH aqueous solutions. On top of the evident superior extraction performance, it should be added that recent advances in the preparation of DES compounds, such as FA, have allowed their sourcing from natural resources using eco-friendly approaches [37]. Extraction with H_2_O represented the lowest concentration of polyphenols recovered in both pine wastes. Finally, it is also observed that the concentration of polyphenols extracted from pine bark was higher than from the needles. This result is aligned with the fact that bark is known to have a higher native concentration of polyphenols [3]. Overall, data suggest that DESs mixtures are an interesting alternative to traditional chemical solvents for the extraction of polyphenols [38,39,40].

One-way ANOVA tests were carried out, obtaining *p*-values of 5.0 × 10^−6^ and 1.3 × 10^−5^ for pine needles and pine bark, respectively, thus ensuring significant differences among the solvents used. After this initial screening, the LA:FA mixture was selected as the DESs mixture for the optimization using the USE-based method for the extraction of polyphenols from pine residues.

### 3.2. BBD-RSM Optimization

Once the DESs solvent was selected for the extraction, the USE method was optimized using a BBD-RSM design where six variables were selected as described in the experimental section. The selection of these variables as well as their range of study were based on the previous experience of the group. In addition, since it was previously observed that the concentration of polyphenols was lower in the pine needle residue than in the bark, it was decided to perform the optimization of the method using only the pine needles (poorer matrix) and later apply the optimized conditions to the pine bark.

This design resulted in a total of 54 experiments (Table 1) that were randomly performed. The extracts obtained were manipulated as previously described. The results were analyzed by means of the BBD-RSM design using the Statgraphics software.

The total polyphenols concentration predicted by the response surface methodology using developed coefficients based on the conditions of each experiment and Equation (1), was compared with the concentration of total polyphenols observed, obtaining an average error of 5.11%. It should be mentioned that this error ranged from 0.11% to 18.29%, being less than 10% in most cases and, therefore, demonstrating the suitability of the developed method. In addition, the R-Squared statistic indicates that the developed model explains 80.73% of the variance of THE response variable, demonstrating its correct mathematical adjustment. The standard error of the estimate shows that the standard deviation of the residuals is 43.71. On the other hand, the Durbin–Watson statistic tests the residuals to determine if there is any significant connection based on the order in which the data is presented. Since the *p*-value is greater than 5.0%, there is no indication of serial autocorrelation in the residuals at the 95% level of confidence.

**Figure 2:**
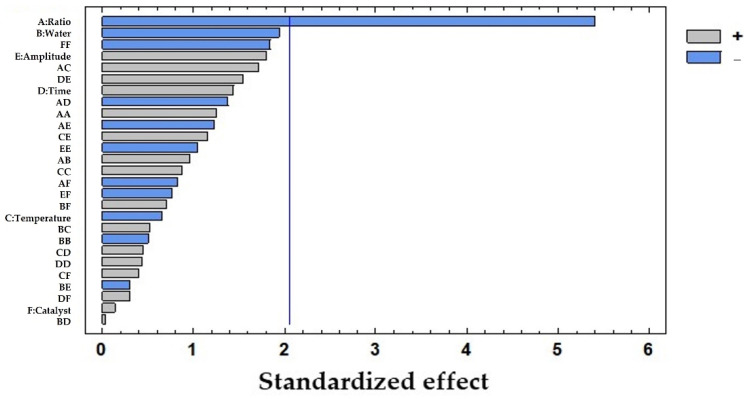
Pareto chart results from the total polyphenols extracted (mg/g dw) from pine needles using USE: A: solid-to-solvent ratio (g sample in 10 mL solvent); B: water (%) in solvent; C: temperature of extraction (°C); D: time of extraction (h); E: amplitude (%); F: catalyst concentration of H_2_SO_4_ (M). The vertical line marks 95% of confidence.

To evaluate the influence of each of the studied variables, an ANOVA test was carried out with LSD considering a 95% confidence level (Table 2). The solid-to-solvent ratio was found to be the only influential variable (*p*-value: 0.0000), while the rest of the variables showed *p*-values > 0.05, with the percentage of water being the closest to being influential (*p*-value: 0.0638). The results were graphically represented in a Pareto diagram as shown in Figure 2. In this chart, the negative influence of the solid-to-solvent ratio on the extraction of polyphenols from pine needles is observed which suggests that the lower the ratio, the higher the concentration of polyphenols extracted.

Finally, since the β coefficients have been calculated using the least-squares method, the response surface methodology is used to obtain the values of the controllable parameters (*X_i_*) that optimize the response or to discover what values will result in the maximum recovery of polyphenols. In this case, the optimal extraction conditions were established as 0.50 g of sample in 10 mL of LA:FA (70:30%, *v*/*v*) with 0% water and 0 M H_2_SO_4_ heated to 30 °C and extracted for 30 min with an amplitude of 80% at 37 kHz.

As can be observed, the amount of sample was found at the lower end of the studied range, and thus it was decided to further expand it. The optimum temperature value was also found at the lower end of the studied range. Nevertheless, to save energy and guarantee better control over the method and repeatability, the lowest temperature is preferred. Therefore, the subsequent trials were performed at 30 °C. On the other hand, the ultrasound amplitude was found at the upper end of the studied range, but given the system used, it was technically impossible to increase this value. Moreover, since it is not an influential variable, it was decided to proceed with the trials with 80% amplitude. Finally, the extraction time was also at the upper end of the studied range, and thus it was decided to further widen the range of this variable as well.

#### Two-Factorial Design

As indicated above, some variables were selected to be further optimized considering a wider range of studies. This was the case of the extraction time and solid-to-solvent ratio. For that, a two-factorial design with RSM was selected to evaluate the influence of the new ranges in the extraction of total polyphenols from pine needles by USE. The new solid-to-solvent ratio range was set from 0.1 to 0.5 g per 10 mL of solvent, while the extraction time was set from 5 to 60 min, implying a total of nine experiments (Table 3) that were randomly performed.

**Figure 3:**
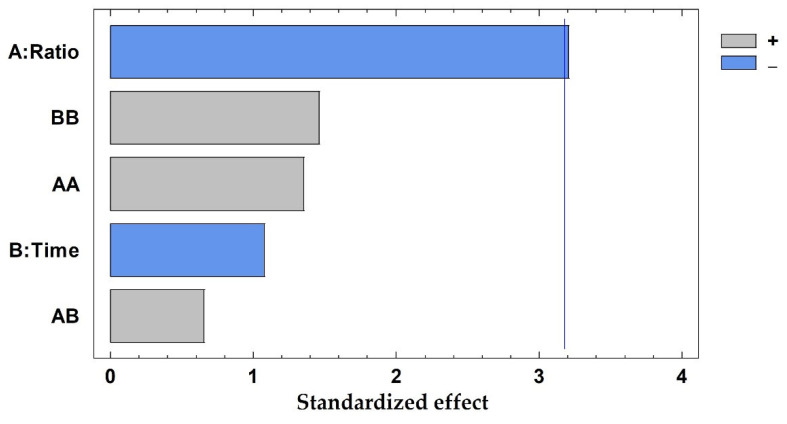
Pareto chart results from the total polyphenols extracted (mg/g dw) from pine needles using factorial design, A: solid-to-solvent ratio (g sample in 10 mL solvent); B: time of extraction (minutes). The vertical line marks 95% of confidence.

The average prediction error was 7.24%, ranging from 0.47% to 18.63%, being lower than 20% in all cases. In addition, the R^2^ coefficient was 0.9333, ensuring a good adjustment of the developed method. The solid-to-solvent ratio was the only influential variable considering a 95% of confidence (*p*-value: 0.0093), whereas the time of extraction and the interaction between variables exhibited *p*-values higher than 0.05, proving that these are not influential in the current method. Results have been graphically represented in a Pareto chart (Figure 3), where the negative influence of the solid-to-solvent ratio can be observed, which means that the highest concentration of polyphenols was achieved when the lowest value of this parameter was considered.

After this extra optimization, the optimal extraction conditions were set at: 0.1 g of sample in 10 mL of LA:FA (70:30%, *v*/*v*) with 0% water and 0 M H_2_SO_4_ heated to 30 °C and extracted for 40 min with an amplitude of 80% at 37 kHz. As can be observed, the amount of sample continued to be in the lowest value of the studied range. However, it was considered that lower amounts of the sample could produce higher errors during the weighting procedure.

It should be highlighted that the optimal extraction conditions agreed with other studies in related residues. Meullemiestre et al. [8] optimized the application of USE for polyphenols extraction from pine sawdust and observed that an extraction performed at 40 °C and 40 min with 40 kHz of power maximizes the extraction yield, i.e., 224 mg/g. Aspé et al. [41] also evaluated the use of an ultrasonic bath in the extraction of polyphenols from *P. radiata* bark, obtaining the highest yield (12.7 mg/g) when 35 kHz at 85 W and 25 °C were used. Lastly, Nisca et al. [42] studied the use of ultrasound in the extraction of polyphenols from *P. nigra* and *P. sylvestris* bark, obtaining the best results (53.41 and 43.05 mg/g, respectively) when 1 g of sample was treated with an alcoholic solution and kept in a bath at 65 °C for 30 min. As can be observed, even if the conditions are not fully comparable, the optimal parameters reported in the literature agree with the conditions optimized in the current research. However, it should be highlighted that, in all cases, the total amount of obtained polyphenols is lower than the ones obtained in the present research. The innovative combination of DES and USE considerably boosts the total concentration of polyphenols recovered from pine biomass.

### 3.3. Repeatability and Intermediate Precision

The repeatability and intermediate precision as parameters were considered to access the suitability of the developed extraction method and the applicability in pine bark residues [43]. Six extractions were carried out under optimal conditions on the same day for the repeatability assay whereas 6 more extractions were performed on 2 consecutive days for the intermediate precision analysis, resulting in a total of 18 extractions. Note that these experiments were applied to both pine needle and bark residues.

Again, the extracts were analyzed at 280 nm to estimate the total concentration of polyphenols, and the coefficient of variation (CV) was the selected variable to infer on the suitability of the method (Table 4). In the repeatability assay, the CV was 1.65% and 1.64%, while in the case of the intermediate precision test, the CV was 3.92% and 3.36% for pine needles and bark residues, respectively. In all cases, the CV was lower than 5%, proving the suitability of the developed method for the superior extraction of polyphenols from pine needles and bark waste. In addition, the average total concentration of polyphenols extracted under optimal conditions from pine needles and bark were 274.38 and 314.62 mg/g, respectively, which are considerably higher than the concentrations previously reported in related studies [8,41,42].

### 3.4. Bioactive Properties of the Extracts

Once polyphenol-enriched extracts from pine needle and bark were obtained under optimal conditions, the following step focused on evaluating their bioactive properties, particularly the antioxidant and antimicrobial activities. The antioxidant activity was evaluated according to the DPPH method [36,44]. For that, three extracts from pine needle and bark obtained using optimal conditions were selected, and the antioxidant activity was estimated following the procedure described in the methodology section. The antioxidant activity of the solvent was also assessed under the same conditions and its contribution subtracted.

The results showed average antioxidant activities of ca. 45.90 ± 2.10 and 66.96 ± 2.75 mg Trolox equivalents/g dw for pine needles and bark, respectively. These results are in perfect agreement with those reported by Nisca et al. [42] where polyphenols extracted with an alcoholic solution with USE exhibited 74.59% and 70.36% inhibition for *P. nigra* and *P. sylvestris* residues, respectively.

**Figure 4:**
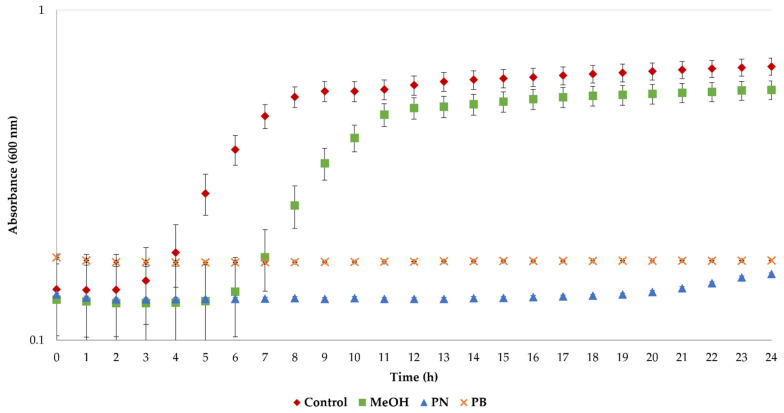
Antimicrobial activity of the extracts from pine needle and bark residues obtained under optimal extraction conditions.

Bacterial cultures were also exposed to pine needle and bark extracts to evaluate their antimicrobial activity. As was previously mentioned, the antimicrobial properties of LA have been previously described [45], and therefore the extracted polyphenols were redissolved in MeOH to avoid any LA effect. As can be observed in Figure 4, the growth curves of *S. aureus* cells display typical sigmoidal profiles for the control cultures. In the case of the solvent (MeOH), the growth of cells was delayed, but exponential growth was achieved after 11 h. More importantly, no growth was observed when the bacteria were exposed to the polyphenols’ extracts, thus demonstrating their remarkable antimicrobial properties.

### 3.5. Solvent Recyclability and Reuse

From economic and environmental perspectives, solvent recyclability and reusability are highly desirable for sustainable large-scale applications. Therefore, the possibility of recycling and reusing LA from the polyphenols-enriched extracts was also evaluated. The extracts were treated as described in the Section 2, allowing the total separation of polyphenols from the solvent based on the similar polarity of Strata-X cartridges and polyphenols. Such favorable interactions induce the polyphenol adsorption and retention in the cartridges and consequently their separation from the solvent. After the evaporation, the recovery yield of LA was ca. 60%.

The recovered solvent was mixed with neat FA to prepare a new DES mixture (70:30%, LA:FA) which was subsequently employed for the extraction of polyphenols in both pine needle and bark under optimal conditions. Each extraction was performed in triplicate. Although the solvent recovery and purification protocols were not optimized and targeted in this work, it was observed that such simple proof of concept has allowed the preparation of novel DES with remarkable extraction capacity, i.e., the extraction performance with the recycled LA has only decreased 8% when compared with neat LA. These successful initial trials demonstrate the feasibility of recycling LA in an accessible way without significantly compromising the extraction performance.

## 4. Conclusions

In the present investigation, the use of novel deep eutectic solvents was evaluated as a sustainable alternative to the traditional chemical solvents commonly used for the extraction of bioactive compounds. Specifically, this study has focused on the extraction of polyphenols, bioactive compounds that have properties of great interest, such as antioxidant, antimicrobial, and anti-inflammatory activities. In addition, these compounds have been extracted from forest residues that are currently being discarded without any added value. Therefore, the possibility of using green solvents for the extraction of these compounds with similar or even higher yields than those observed in the literature has been demonstrated.

The optimization of an ultrasound-assisted extraction method was performed by targeting the recovery of polyphenols. It was found that the solid-to-solvent ratio (*p*-value: 0.000) is the only significantly influential variable in the process, and the following optimal conditions were established: 0.1 g of pine residue in 10 mL of LA:FA (70:30%, *v*/*v*) with 0% water and 0 M H_2_SO_4_ heated to 30 °C and extracted 40 min with ultrasound amplitude of 80% at 37 kHz. The suitability of the method was demonstrated by reaching coefficients of variation lower than 5% when the repeatability and intermediate precision assays were evaluated. Although not fully optimized, solvent recyclability and reusability were shown to be possible; separation of the bioactive compounds from the solvent used was achieved with a simple and affordable procedure, where more than 60% of levulinic acid was recovered and successfully reused in new extractions.

Finally, the remarkable antioxidant and antimicrobial properties of the polyphenol-enriched pine extracts were demonstrated. This represents an important scientific advance toward sustainable alternatives for the extraction of added-value bioactive compounds with potential applications in different fields, such as nutrition, medicine, or nutraceutical. We believe that the combination of DES with the USE approach described in this work results in a novel and attractive green extraction process promising for large-scale applications, thus strongly contributing to the valorization of forest-based residues into novel end-applications.

## Figures and Tables

**Table 1:** Box–Behnken design conditions used for the optimization of the USE-based extraction of polyphenols from pine needles.

Experiment	Solid-to-Solvent Ratio (g/10 mL Solvent)	Water (%)	Temperature (°C)	Time (h)	Amplitude (%)	Catalyst (M)	Total Polyphenols Observed (mg/g dw)	Total Polyphenols Adjusted (mg/g dw)	Error (%)
1	2.5	30	45	30.0	80	1	58.77	60.33	2.66
2	1.5	60	45	17.5	80	0	31.05	30.09	3.08
3	2.5	30	45	30.0	20	1	20.72	18.45	10.97
4	1.5	30	45	17.5	50	1	80.31	81.08	0.95
5	0.5	30	60	17.5	50	0	81.39	82.49	1.35
6	2.5	30	30	17.5	50	0	32.14	32.21	0.23
7	1.5	0	45	17.5	20	0	28.37	30.51	7.55
8	1.5	0	45	17.5	80	0	91.24	93.06	1.99
9	1.5	30	30	5.0	50	0	81.18	81.58	0.50
10	1.5	0	60	17.5	20	1	39.91	38.02	4.73
11	0.5	30	45	5.0	80	1	103.62	121.54	17.29
12	1.5	30	30	30.0	50	0	82.21	83.98	2.15
13	1.5	30	45	17.5	50	1	77.90	81.08	4.08
14	1.5	30	60	5.0	50	0	48.57	47.12	2.98
15	1.5	0	60	17.5	80	1	137.89	112.67	18.29
16	0.5	30	45	5.0	20	1	114.86	98.94	13.86
17	1.5	30	45	17.5	50	1	82.88	81.08	2.18
18	1.5	0	30	17.5	20	1	118.36	101.59	14.17
19	0.5	30	45	30.0	20	1	102.27	107.00	4.63
20	1.5	60	60	17.5	80	1	84.73	87.57	3.36
21	1.5	30	45	17.5	50	1	78.48	81.08	3.31
22	2.5	60	45	5.0	50	1	52.29	48.16	7.91
23	0.5	0	45	30.0	50	1	205.00	204.78	0.11
24	1.5	30	45	17.5	50	1	89.24	81.08	9.15
25	1.5	30	30	30.0	50	2	87.33	86.91	0.48
26	0.5	30	60	17.5	50	2	121.38	119.44	1.60
27	2.5	30	30	17.5	50	2	49.45	49.01	0.89
28	2.5	30	45	5.0	20	1	74.31	70.56	5.05
29	0.5	30	30	17.5	50	0	189.79	155.78	17.92
30	1.5	60	45	17.5	20	2	34.47	28.73	16.64
31	1.5	60	45	17.5	80	2	28.89	30.67	6.16
32	2.5	30	60	17.5	50	0	65.28	64.64	0.99
33	0.5	30	30	17.5	50	2	162.77	175.28	7.68
34	0.5	0	45	5.0	50	1	166.03	150.24	9.51
35	1.5	30	60	5.0	50	2	49.04	49.14	0.19
36	1.5	30	60	30.0	50	0	83.67	77.57	7.29
37	2.5	0	45	5.0	50	1	53.86	54.31	0.83
38	1.5	30	60	30.0	50	2	86.50	97.96	13.25
39	2.5	0	45	30.0	50	1	42.64	48.68	14.16
40	1.5	60	60	17.5	20	1	26.76	26.34	1.57
41	1.5	60	30	17.5	20	1	58.43	57.58	1.46
42	0.5	60	45	5.0	50	1	86.66	84.98	1.94
43	1.5	60	30	17.5	80	1	49.45	47.42	4.11
44	2.5	30	60	17.5	50	2	47.93	50.08	4.49
45	1.5	60	45	17.5	20	0	35.10	35.01	0.26
46	2.5	60	45	30.0	50	1	44.69	44.83	0.32
47	1.5	30	45	17.5	50	1	77.64	81.08	4.42
48	1.5	0	45	17.5	80	2	50.13	50.22	0.19
49	1.5	30	30	5.0	50	2	71.91	66.14	8.02
50	2.5	30	45	5.0	80	1	17.55	17.17	2.15
51	0.5	60	45	30.0	50	1	146.63	141.83	3.28
52	1.5	0	30	17.5	80	1	107.50	104.84	2.47
53	1.5	0	45	17.5	20	2	34.78	34.88	0.28
54	0.5	30	45	30.0	80	1	226.76	224.87	0.83

**Table 2:** Variance analysis of the quadratic model stepwise adjusted to the USE-based extraction of polyphenols from pine needles.

Variable	Sum of Squares	*F*-Value	*p*-Value
A: Solid-to-solvent ratio	55,828.20	29.21	0.0000
B: Water %	7163.94	3.75	0.0638
C: Temperature	822.16	0.43	0.5177
D: Time	3934.98	2.06	0.1632
E: Amplitude	6236.83	3.26	0.0824
F: Catalyst	36.70	0.02	0.8908
AA	3008.53	1.57	0.2208
AB	1747.00	0.91	0.3478
AC	5586.77	2.92	0.0992
AD	3620.43	1.89	0.1804
AE	2886.48	1.51	0.2301
AF	1326.38	0.69	0.4124
BB	481.22	0.25	0.6200
BC	522.78	0.27	0.6054
BD	2.67	0.00	0.9705
BE	179.76	0.09	0.7615
BF	942.21	0.49	0.4888
CC	1475.29	0.77	0.3877
CD	393.54	0.21	0.6537
CE	2548.62	1.33	0.2587
CF	304.50	0.16	0.6930
DD	356.53	0.19	0.6694
DE	4538.19	2.37	0.1354
DF	168.82	0.09	0.7687
EE	2076.20	1.09	0.3069
EF	1113.92	0.58	0.4521
FF	6499.48	3.40	0.0766
Total Error	49,689.10		

**Table 3:** Experimental conditions and the resulting two-factorial design used to optimize: solid-to-solvent ratio and extraction time parameters.

Experiment	Solid-to-Solvent Ratio (g/10 mL Solvent)	Time (min)	Total Polyphenols Observed (mg/g dw)	Total Polyphenols Adjusted (mg/g dw)	Error (%)
1	0.50	5.00	87.76	91.40	4.14
2	0.50	32.50	126.71	126.11	0.47
3	0.30	60.00	151.78	145.60	4.07
4	0.30	32.50	138.01	163.72	18.63
5	0.50	60.00	104.55	101.51	2.91
6	0.10	32.50	280.01	254.90	8.97
7	0.10	60.00	234.03	243.25	3.94
8	0.10	5.00	191.33	207.22	8.31
9	0.30	5.00	142.06	122.53	13.75

**Table 4:** Repeatability (*n* = 6) and intermediate precision (*n* = 18) tests for polyphenols recovery from pine needles and bark residues.

		Average (mg/g dw)	Standard Deviation	CV (%)
Pine Needle	Repeatability	266.87	4.41	1.65
	Intermediate precision	274.38	10.75	3.92
Pine Bark	Repeatability	315.50	5.18	1.64
	Intermediate precision	314.62	10.57	3.36

## Data Availability

Data is contained within the article.
